# Reply to “How to Prevent Arrhythmias Following Acute Coronary Syndrome”

**DOI:** 10.1002/clc.70098

**Published:** 2025-02-13

**Authors:** Luca Cumitini, Ailia Giubertoni, Giuseppe Patti

**Affiliations:** ^1^ Department of Translational Medicine University of Eastern Piedmont Novara Italy; ^2^ Division of Cardiology Maggiore Della Carità Hospital Novara Italy

We would like to thank Naoya Kataoka and Teruhiko Imamura for their thoughtful comments regarding our clinical research, and we would like to take the opportunity to point out some aspects.

We fully agree that including additional predictors, such as hyperuricemia, chronic obstructive pulmonary disease, and specific electrocardiographic/echocardiographic parameters may potentially improve the ability of detecting atrial fibrillation (AF) and ventricular arrhythmias (VA) [1]. However, in our study we focused on the application of the PRAISE (PRedicting with Artificial Intelligence riSk aftEr acute coronary syndrome) score model [2] to ensure consistency with the original validation and clinical interpretability of the results. This approach allowed us to evaluate the performance of the model in a real‐world context without introducing additional variables that, if incorporated in the score, would have required its recalibration and revalidation. We recognize that the addition of these factors may be an interesting direction for future research.

Anemia increases the risk of arrhythmias [3], alongside other variables, such as older age and reduced left ventricular ejection fraction (LVEF). In our clinical research, at univariate analysis LVEF was associated with the development of AF and VA, and age with the occurrence of VA. However, at multivariate analysis LVEF and age were not independent predictors of early arrhythmic complications [4]. Moreover, we believe that the comprehensive use of the PRAISE score overcomes the inherent limitations of stratification based solely on traditional, individual parameters.

Finally, we found that the PRAISE score is a machine learning‐based risk stratification tool with high specificity for predicting arrhythmic complications during hospitalization. By identifying patients at elevated risk for arrhythmias, the PRAISE score can allow for targeted interventions, such as enhanced rhythm monitoring or optimizing pharmacological treatments. These strategies are particularly relevant within the first 30 days postacute coronary syndrome (ACS), that is, during the critical phase of cardiac reverse remodeling. We demonstrated an early predictive value of the PRAISE score for arrhythmias, but its role in guiding long‐term interventions remains under investigation [4]. Incorporating the PRAISE score into post‐ACS management could potentially improve outcomes even over the longer‐term, by facilitating early detection of high‐risk patients, promoting tailored use of secondary prevention measures (e.g., intensive lifestyle modifications, stricter rhythm monitoring, or extended pharmacological therapy), and optimizing resource allocation for arrhythmia prevention. However, this needs to be evaluated in future, prospective protocols. Further studies are also welcome to evaluate the utility of the PRAISE score in selecting patients candidates to wearable cardioverter‐defibrillators in the early phase post‐ACS and its potential to refine timing and necessity of implantable devices in the chronic phase.

## Conflicts of Interest

The authors declare no conflicts of interest.

## Data Availability

Data are available on request due to privacy/ethical restrictions. The data that support the findings of this study are available on request from the corresponding author. The data are not publicly available due to privacy or ethical restrictions.
